# Longitudinal Changes of CT-radiomic and Systemic Inflammatory Features Predict Survival in Advanced Non–Small Cell Lung Cancer Patients Treated With Immune Checkpoint Inhibitors

**DOI:** 10.1097/RTI.0000000000000801

**Published:** 2024-11-25

**Authors:** Maurizio Balbi, Giulia Mazzaschi, Ludovica Leo, Lucas Moron Dalla Tor, Gianluca Milanese, Cristina Marrocchio, Mario Silva, Rebecca Mura, Pasquale Favia, Giovanni Bocchialini, Francesca Trentini, Roberta Minari, Luca Ampollini, Federico Quaini, Giovanni Roti, Marcello Tiseo, Nicola Sverzellati

**Affiliations:** * Unit of Scienze Radiologiche; ‡ Medical Oncology Unit; ∥ Translational Hematology Unit; § Thoracic Surgery Unit, University Hospital of Parma; † Department of Medicine and Surgery, University of Parma, Parma, Italy

**Keywords:** immune checkpoint inhibitors, prognosis, carcinoma, non–small cell lung, biomarkers, cancer, multidetector computed tomography

## Abstract

**Purpose:**

This study aims to determine whether longitudinal changes in CT radiomic features (RFs) and systemic inflammatory indices outperform single-time-point assessment in predicting survival in advanced non–small cell lung cancer (NSCLC) treated with immune checkpoint inhibitors (ICIs).

**Materials and Methods:**

We retrospectively acquired pretreatment (T0) and first disease assessment (T1) RFs and systemic inflammatory indices from a single-center cohort of stage IV NSCLC patients and computed their delta (Δ) variation as [(T1-T0)/T0]. RFs from the primary tumor were selected for building baseline-radiomic (RAD) and Δ-RAD scores using the linear combination of standardized predictors detected by LASSO Cox regression models. Cox models were generated using clinical features alone or combined with baseline and Δ blood parameters and integrated with baseline-RAD and Δ-RAD. All models were 3-fold cross-validated. A prognostic index (PI) of each model was tested to stratify overall survival (OS) through Kaplan-Meier analysis.

**Results:**

We included 90 ICI-treated NSCLC patients (median age 70 y [IQR=42 to 85], 63 males). Δ-RAD outperformed baseline-RAD for predicting OS [c-index: 0.632 (95%CI: 0.628 to 0.636) vs. 0.605 (95%CI: 0.601 to 0.608) in the test splits]. Integrating longitudinal changes of systemic inflammatory indices and Δ-RAD with clinical data led to the best model performance [Integrated-Δ model, c-index: 0.750 (95% CI: 0.749 to 0.751) in training and 0.718 (95% CI: 0.715 to 0.721) in testing splits]. PI enabled significant OS stratification within all the models (*P*-value <0.01), reaching the greatest discriminative ability in Δ models (high-risk group HR up to 7.37, 95% CI: 3.9 to 13.94, *P*<0.01).

**Conclusion:**

Δ-RAD improved OS prediction compared with single-time-point radiomic in advanced ICI-treated NSCLC. Integrating Δ-RAD with a longitudinal assessment of clinical and laboratory data further improved the prognostic performance.

Inhibition of either programmed death protein 1 (PD-1) or programmed death-ligand 1 (PD-L1), otherwise known as immune checkpoint inhibitors (ICIs), has led to remarkable therapeutic improvements in advanced non–small cell lung cancer (NSCLC), yet only a subset of patients achieves a meaningful and durable survival benefit.^1–4^ This scenario advocates for biomarkers capable of optimizing NSCLC patient selection, ideally enabling the identification of cases in which survival gain may be maximized.^5^

Among noninvasive approaches for cancer patient management, the conversion of digital medical images into mineable high-dimensional data, a process defined as radiomics, has shown promise to play a role in the characterization and monitoring of lung cancer.^6,7^ Most studies exploring radiomics in NSCLC have focused on a single-time approach to predict outcomes such as histology, tumor PD-L1 expression and genomics, disease response, and overall survival (OS).^8–12^ Delta-radiomics (ΔR) assesses the changes in radiomic features (RFs) over time and aims to decipher NSCLC behavior beyond size modification.^13^ This information may be particularly relevant in patients treated with ICIs, in whom a discrepancy between tumor size and disease response is a recognized occurrence.^14^ While preliminary evidence suggests that ΔR may help predict response to ICIs in NSCLC,^15–19^ only a few studies have evaluated ΔR for survival stratification and compared it with the baseline in this setting, revealing potential for longitudinal models to overcome those based on pretreatment CT alone.^16,18,20^

Peripheral blood may represent a source of bio-humoral and cellular parameters to predict response to treatment in cancer patients. Indeed, different systemic inflammatory indices, such as neutrophils,^21,22^ lymphocytes, and lactate dehydrogenase (LDH), have demonstrated prognostic impact in several malignancies.^23^ A series of investigations have pointed attention toward the derived neutrophil to lymphocyte ratio (dNLR) and LDH levels, constituting Lung Immune Prognostic Index, as robust predictors of NSCLC outcome following chemo, target, and, above all, ICI therapy.^24–26^

In the present study, we aimed to compare the prognostic value of longitudinal changes in both radiomics and laboratory data comprehensive of systemic inflammatory parameters with single-time-point assessment for predicting survival in advanced NSCLC patients treated with ICIs.

## MATERIALS AND METHODS

### Patient Population and Study Design

**FIGURE 1:**
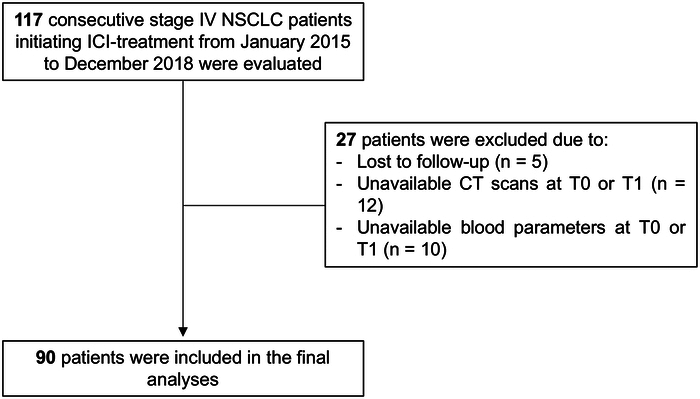
Consort flow diagram for patient selection.

**FIGURE 2:**
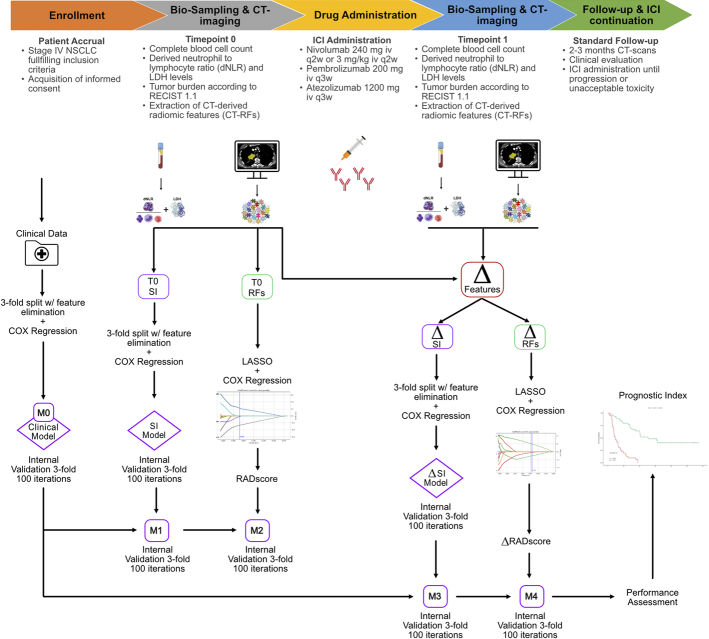
Overall study design, including timeline, data collection, radiomic analysis and models development.

All the non–oncogene-addicted stage IV NSCLC patients who initiated ICIs from January 2015 to December 2018 at the Medical Oncology Unit of the University Hospital of Parma (Italy) were considered for inclusion in the present study. In particular, out of the initially selected 117 consecutive NSCLC patients, 27 (23%) were excluded due to the following exclusion criteria: (a) active pneumonitis at the time of pretreatment (T0) or first post-treatment (T1) CT scan; (b) lack of T0 or T1 evaluations comprehensive of CT imaging, LDH or dLNR; (c) unavailability of clinical, laboratory, or imaging data, including follow-up (Fig. 1). Hence, the final study population included 90 NSCLC patients with a median age of 70 years (IQR=42 to 85), a predominance of males (63/90, 70%), and a proportion of current and former smokers of, respectively, 29% (26/90) and 37% (33/90). All these patients underwent CT and clinical laboratory investigations, including Eastern Cooperative Oncology Group (ECOG) performance status, clinical stage, and peripheral blood sampling at T0 and T1, as outlined in Figure 2.

The study was performed following approval from the Local Ethical Committee, which waived the need for written informed consent.

### Image Acquisition and Tumor Burden Evaluation

T0 and T1 CT examinations were acquired within 1 week from the respective clinical laboratory assessment using various multidetector CT scanners (SOMATOM Definition Flash, SOMATOM Definition Edge, SOMATOM Sensation 64 Cardiac, SOMATOM Emotion 6; Siemens Healthineers, Erlangen, Germany). All the acquisitions were performed following contrast medium administration. Parameters of the imaging series retrieved for the analyses were as follows: slice thickness, 1.5 to 2.5 mm; tube voltage, 100 to 120 kVp; automated exposure control for tube current; contiguous or overlap reconstruction interval; smooth kernels.

The tumor burden (TB) computed by consultant radiologists in charge following the RECIST 1.1 criteria^27^ was retrospectively collected from the electronic medical records for the purposes of the current analyses.

### Image Processing and Radiomic Feature Extraction

The postcontrast CT images were uploaded to a dedicated workstation for processing and feature extraction. For each examination, a volumetric segmentation of the primary tumor was performed by a 5-year experienced thoracic radiologist blinded to clinical data other than the tumor location. All the segmentations were performed semiautomatically using open-source software (3D Slicer, Version 4.11), adjusting the window width and level to properly annotate tumor borders, excluding bronchi, blood vessels, vacuoles, normal lung tissue, chest wall, and mediastinal structures. A total of 25 randomly selected tumors were also segmented by a 3-year experienced thoracic radiologist to evaluate the feature robustness. The volumes of interest were resampled to 1x1x1-mm voxel size using linear interpolation to counter different slice thicknesses. Moreover, signal intensity values were discretized to a bin width of 25 to further reduce variability. RFs were extracted using the open-source tool pyRadiomics integrated as a plugin into 3D Slicer software.

### Peripheral Blood Systemic Inflammatory Indices

The LDH levels were measured on Beckman Coulter AU5800 using the International Federation of Clinical Chemistry-recommended procedure and reported as unit/litre. Values resulting within the normal range (0 to 248 U/L) or higher than the upper limit of normality (> UNL) were separately considered. Complete blood cell count and leukocyte differential were obtained by automated routine hemocytometric analysis using Advia 2120 (Siemens Healthcare Diagnostics, Tarrytown, NY) according to the manufacturer's instructions. The LDH and dNLR (absolute neutrophil count / [white blood cell concentration−absolute neutrophil count]), collectively defined hereafter as systemic inflammatory indices, were manually computed at T0 and T1.

### Model Description

We developed survival models combining radiomic signatures and clinical laboratory data comprehensive of systemic inflammatory indices. The RFs were selected by a penalized Cox regression model and combined to obtain radiomic scores. In parallel, we designated informative clinical and laboratory variables to fit into Cox regression models to be integrated with the radiomic scores, and we evaluated the performances for OS stratification. This approach was adopted for baseline data and dynamic variables for whom longitudinal changes could be assessed.

### Radiomic Features Selection and Models’ Development

The Spearman rho correlation coefficient was performed to remove redundant T0 and T1 RFs, that is, variables with a coefficient >0.99 were excluded. Moreover, only RFs showing an intraclass correlation coefficient (ICC) ≥0.65 from the 25 scans segmented by the 2 readers were maintained to ensure sufficient stability. The resulting RFs constituted the T0 and T1 data sets. A delta data set was then created by calculating the longitudinal variation of the selected RFs as [T1-T0]/T0.

LASSO Cox OS models were trained and validated on the T0 and the delta data set to develop a baseline-radiomic (RAD) and a delta-radiomic (Δ-RAD) model, respectively, using the following steps. We first explored 100 different values of penalty strengths, setting the number of iterations at 100. Within this set of estimated penalties, a 3-fold cross-validation was performed to calculate the performance of each explored penalty value in terms of concordance index (c-index). Finally, we selected the best penalty strength that provided the best *c*-index performance and set this configuration as the final model. By using this latter model, we iterated a 3-fold cross-validation 100 times to calculate the mean and 95% CIs of the *c*-index in the train and test splits. For each iteration, both train and test data underwent *Z*-score standardization. RAD-score and Δ-RAD-score were computed using the linear combination of standardized predictors selected by the final baseline-RAD and Δ-RAD model weighted on the basis of their respective coefficients, in keeping with the previous methodology.^28^

All analyses were performed in Python 3.9.12 using the scikit-survival library for the survival analysis.

### Clinical Laboratory and Integrated Models’ Development

The most significant predictors among systemic inflammatory indices and TB were selected by performing Cox regression analysis through 3-fold cross-validation with backward feature elimination. The Akaike Information Criterion was employed as a stopping rule. We applied the same strategy to select baseline clinical features to fit into a Cox model (M0) and then combined it with the optimal sets of laboratory data. In particular, 2 Cox models were obtained by combining M0 with baseline systemic inflammatory indices (M1) and both delta-TB (Δ-TB) and delta-LDH (Δ-LDH) (M3). Moreover, we generated 2 fully integrated Cox models by adding baseline-RAD and Δ-RAD scores to M1 and M3 models leading, respectively, to an integrated baseline M2 and an integrated-Δ M4 Cox model. All these models were internally validated using a repeated (100 iterations) 3-fold cross-validation strategy. Splitting the data into training and test sets allowed us to compute mean *c*-indexes for each training and test model, and their 95% CIs were used for model description and performance.

### Prognostic Index

A prognostic index (PI) was computed as a linear combination of variables and their Cox coefficients to build stratified Kaplan-Meier curves for each model. The median PI value of each Cox model was used as a threshold to create 2 categories describing high and low PI. Furthermore, for each model, a new Cox model was generated using the respective PI to obtain the overall hazard ratio, likelihood ratio, and overall Cox *P*-value.

### Statistical Analysis

The primary endpoint of this study was OS, defined as the time from ICI start until death from any cause as of September 30, 2021. The Cox proportional hazard regression model was used to adjust for relevant clinicopathologic variables in multivariable analysis. Kaplan-Meier analysis and Log-rank tests were used to evaluate the statistical significance of patient stratification by the proposed signatures. The cutoff value of PI was optimized by the Log-rank test based. All statistical tests were 2-sided, with a *P*-value <0.05 considered statistically significant. The analyses were performed by means of R-4.2.2 using rms and survival packages.

## RESULTS

### Patient Population and Laboratory Characteristics

The study population included 90 NSCLC patients, whose demographic and clinical variables are detailed in Table 1.

**TABLE 1:** Patient Population Characteristics

Total, n	90
Age, y	70 [42–85]*
	**n (%)**
Sex	
Male	63 (70)
Female	27 (30)
Smoking status	
Current smokers	26 (29)
Former smokers	33 (37)
Never Smokers	31 (34)
ECOG PS	
0-1	84 (93)
2	6 (7)
Histotype	
SCC	29 (32)
ADC	57 (63)
NSCLC NOS	4 (5)
ICI Treatment	
I line	8 (9)
II line	56 (62)
≥III line	26 (29)
ICI molecule	
Nivolumab	70 (78)
Pembrolizumab	13 (14)
Atezolizumab	7 (8)
Metastatic involvement	
Lymph nodes	83 (92)
Liver	13 (14)
Bone	32 (36)
Adrenal	11 (12)
Brain	13 (14)
Contralateral lung	88 (98)
Pleura	23 (26)
PD-L1 status	
<1%	15 (17)
1%-49%	32 (35)
≥50%	20 (22)
Not tested	23 (26)
Mutational status	
KRAS mut†	15 (38)
EGFR exon 20/ MET/BRAF mut†	9 (23)
Not tested‡	22 (36)

* Data are reported as median [IQR].

† The percentage refers to the number of cases over the entire population of molecularly tested ADC and NSCLC NOS (N=39).

‡ The percentage refers to the number of cases over the entire population of ADC and NSCLC NOS (n=61).

ADC indicates adenocarcinoma; ECOG PS, Eastern Cooperative Oncology Group Performance Status; IQR, interquartile range; ICI, immune checkpoint inhibitors; NSCLC, non–small cell lung cancer; NOS, not otherwise specified; PD-L1, programmed cell death-ligand-1; SCC, squamous cell carcinoma.

Adenocarcinoma represented the most frequent histology (57/90, 63%). Contralateral lung and lymph node metastases were present in more than 90% of stage IV NSCLC patients (88/90, 98%, and 83/90, 92%, respectively), followed by bone (32/90, 36%) involvement. ICIs were given as second or subsequent-line treatment in 62% (56/90) and 29% (27/90) of cases, respectively. Among the 67 tested patients, a PD-L1 tumor proportion score ≥50% was detected in nearly one-third of cases (20/67, 30%), ranging from 1% to 49% in almost half of the patients (32/67, 48%).

With a median follow-up of 30.6 months (95% CI: 17.9 to 43.4), the OS was 10.7 months (95% CI: 6.1 to 14.3), and progression-free survival was 5.1 months (95% CI: 0.8 to 7.5). The average baseline LDH value was 535 U/L, whereas absolute neutrophil count, lymphocyte count, and dNLR were 6246/µL, 1456/µL, and 3.0, respectively.

### Radiomic Models

A total of 107 RFs were initially extracted from the volume of interest of each patient at the 2 time points, including shape, first-order, Gray-Level-Cooccurrence-Matrix (GLCM), Gray-Level-Run-Length-Matrix (GLRLM), Gray-Level-Size-Zone-Matrix (GLSZM), Neighboring-Gray-Tone-Difference-Matrix (NGTDM), and Gray-Level-Dependence-Matrix (GLDM) RFs. After omitting RFs showing high correlations and those with unsatisfactory interobserver reproducibility, 24 T0 and 10 Δ RFs were used for baseline-RAD and Δ-RAD model building, respectively (Table 2). The selected penalty strength of baseline-RAD model (0.131) included *firstorder_maximum, gldm_SmallDependenceLowGrayLevelEmphasis, and ngtdm_Coarseness*, whereas the penalty term of Δ-RAD model (0.066) selected *shape_LeastAxisLength, shape_Maximum3DDiameter*, and *firstorder_Maximum* (Fig. S1, Supplemental Digital Content 1, http://links.lww.com/JTI/A319). Results from 3-fold cross-validation showed mean c-indexes of 0.610 (95% CI: 0.608 to 0.612) and 0.605 (95% CI: 0.601 to 0.608) in the training and test splits, respectively, of baseline-RAD model. The mean *c*-indexes of Δ-RAD model were 0.662 (95% CI: 0.660 to 0.664) in the training and 0.632 (95% CI: 0.628 to 0.636) in the test splits.

**TABLE 2:** Radiomic Features Involved in Baseline-RAD and Δ-RAD Models Building After Preprocessing Steps

Data set	Feature name	Type
T0, Δ	LeastAxisLength	Shape
T0, Δ	Maximum2DDiameterColumn	Shape
T0, Δ	Maximum2DDiameterRow	Shape
T0, Δ	Maximum2DDiameterSlice	Shape
T0, Δ	Maximum3DDiameter	Shape
T0, Δ	MeshVolume	Shape
T0, Δ	MinorAxisLength	Shape
T0, Δ	Maximum	Firstorder
T0, Δ	SizeZoneNonUniformity	GLSZM
T0, Δ	DependenceNonUniformity	GLDM
T0	Flatness	Shape
T0	MajorAxisLength	Shape
T0	Sphericity	Shape
T0	SurfaceVolumeRatio	Shape
T0	10Percentile	Firstorder
T0	Mean	Firstorder
T0	Median	Firstorder
T0	RootMeanSquared	Firstorder
T0	LowGrayLevelRunEmphasis	GLRLM
T0	RunLengthNonUniformity	GLRLM
T0	GrayLevelNonUniformity	GLSZM
T0	LowGrayLevelZoneEmphasis	GLSZM
T0	SmallDependenceLowGrayLevelEmphasis	GLDM
T0	Coarseness	NGTDM

### Integrated Models

**FIGURE 3:**
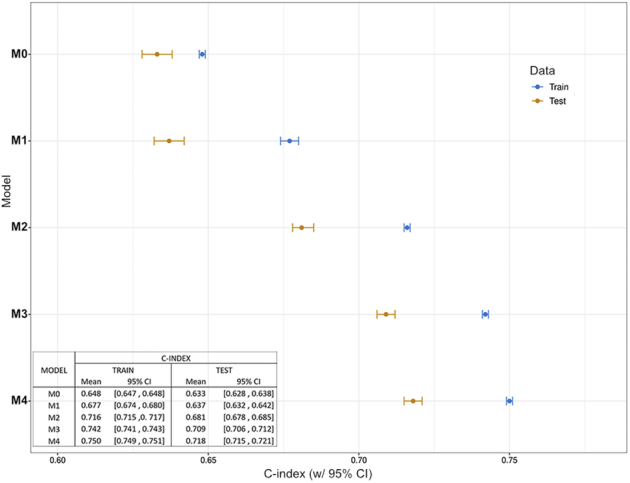
Models’ performances in terms of *c*-index. The plots correspond to the mean and 95% CI of *c*-index distribution in training and test splits. Corresponding numerical values of each individual model are detailed in the inset.

The parameters included in each model and their performances are summarized in Table 3. The performance of the CL model (M0) in the training split increased from a *c*-index of 0.648 (95% CI: 0.647 to 0.648) to 0.677 (95% CI: 0.674 to 0.680) and 0.716 (95% CI: 0.715 to 0.717) when adding SI (M1) and both SI and baseline-RAD score (M2), respectively. The improvement was confirmed in the testing split (M2), reaching a *c*-index of 0.681 (95% CI: 0.678 to 0.685). A higher discriminative ability was reached by integrating information from longitudinal assessment. The integrated-Δ model (M4), in which LDH, but not dNLR, was retained as a significant variable, displayed the highest performance, with a *c*-index of 0.750 (95% CI: 0.749 to 0.751) in the training split and 0.718 (95% CI: 0.715 to 0.721) in the testing split. Integrated models’ performances are summarized in Figure 3.

**TABLE 3:** Models’ Parameters, Including Coefficient, Hazard Ratio (HR), Standard Error (SE) of Coefficient, Wald test With Related *P*-value (Pr(>|Z|)) for Each Feature Included in Cox regression Models

Models	Coef	HR	SE	Wald *Z*	Pr (>|*Z*|)
M0					
Bone lesions	0.741	2.097	0.265	2.800	0.005
ICI line (II) therapy	1.888	6.606	1.016	1.860	0.063
ICI line (III) therapy	1.599	4.947	1.031	1.550	0.121
M1					
Bone lesions	0.658	1.931	0.282	2.340	0.020
ICI line (II) therapy	2.428	11.333	1.071	2.270	0.023
ICI line (III) therapy	2.010	7.465	1.069	1.880	0.060
Basal dNLR	0.250	1.285	0.071	3.540	<0.001
Basal LDH	0.001	1.001	0.000	3.230	0.001
M2					
Bone lesions	0.859	2.362	0.299	2.870	0.004
ICI line (II) therapy	2.181	8.851	1.086	2.010	0.045
ICI line (III) therapy	1.658	5.250	1.087	1.530	0.127
Basal dNLR	0.250	1.285	0.073	3.420	0.001
Basal LDH	0.001	1.001	0.000	2.960	0.003
Baseline-RAD score	3.150	23.329	1.228	2.570	0.010
M3					
Bone lesions	0.657	1.928	0.279	2.350	0.019
ICI line (II) therapy	1.761	5.819	1.016	1.730	0.083
ICI line (III) therapy	1.311	3.711	1.032	1.270	0.204
Delta LDH	0.004	1.004	0.003	1.500	0.133
Delta TB	0.012	1.012	0.003	3.880	<0.001
M4					
Bone lesions	0.795	2.214	0.284	2.800	0.005
ICI line (II) therapy	1.262	3.532	1.034	1.220	0.222
ICI line (III) therapy	0.798	2.221	1.049	0.760	0.447
Delta LDH	0.003	1.003	0.003	1.040	0.297
Delta TB	0.013	1.013	0.003	3.860	<0.001
Δ-RAD score	1.154	3.170	0.442	2.610	0.009

M0 = clinical parameters; M1 = clinical parameters + baseline systemic inflammatory indices; M2 = clinical parameters + baseline systemic inflammatory indices + baseline RAD score; M3 = clinical parameters + delta LDH + delta-TB; M4 = clinical parameters + delta LDH + delta-TB + delta RAD score.

Coef indicates coefficient; dNLR, derived neutrophil to lymphocyte ratio; HR, hazard ratio; ICI, immune checkpoint inhibitor; Pr (>|*Z*|), *P*-value derived from Wald *Z* test; SE, standard error of coefficient; TB, tumor burden.

### Prognostic Index and Survival Outcome

**FIGURE 4:**
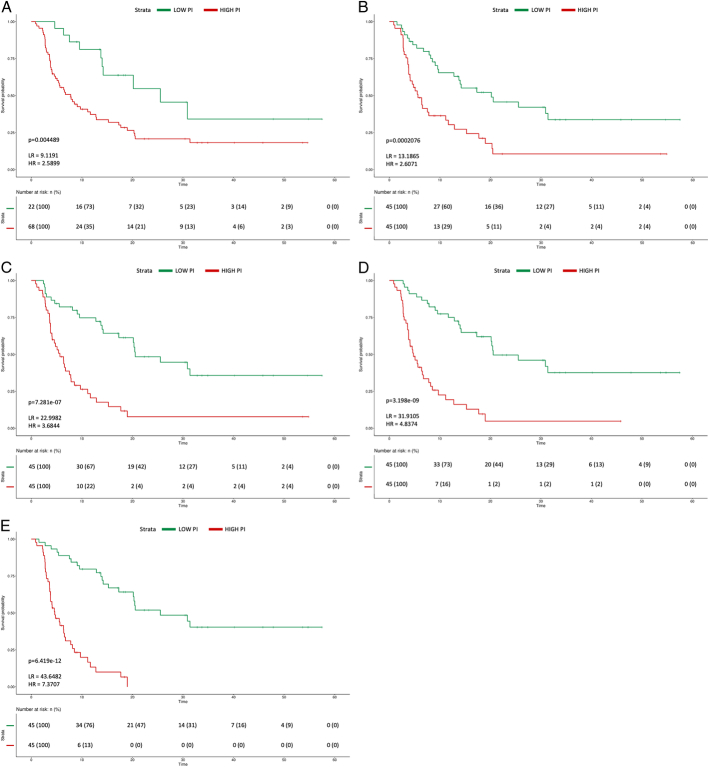
Prognostic index (PI). Kaplan-Meier curves of overall survival (OS) for 2 risk groups identified by PI of each model; hazard ratio, likelihood ratio, and *P*-value derived by Cox regression analysis of each model using the corresponding PI are detailed (A–E).

The patients were stratified into low-risk versus high-risk groups according to PI, which was computed as a linear combination of variables and their Cox coefficients. The PI enabled significant OS stratification within all the models (*P*-value <0.01) (Figs. 4A–E). The greatest discriminative ability was detected for the integrated-Δ (M4) model, in which the high-risk PI group was associated with nearly sevenfold risk of death compared with the lower-risk group (HR=7.37, 95% CI: 3.9 to 13.94, *P*<0.01) (Fig. 4E). In the same model, the median OS resulted significantly longer in the low-risk group (mOS=25.5 mo, 95% CI: 20.16 to NA) compared with high-risk patients (mOS=4.64  mo, 95% CI: 3.78 to 6.74). Scatterplots of OS versus PI are illustrated in Supplementary Figure S2 (Supplemental Digital Content 2, http://links.lww.com/JTI/A320).

## DISCUSSION

The search for robust biomarkers suitable to refine NSCLC prognosis is still ongoing. Generating multivariate risk prediction models based on monitoring of longitudinal imaging, laboratory, and clinical data rather than a single-time-point evaluation may be valuable to this purpose, but evidence in favor of such an approach is still limited. The study findings showed that ΔR was superior to single-time-point radiomic signature in predicting OS in NSCLC patients treated with immunotherapy. This was also true for changes in TB and peripheral blood systemic inflammatory indices.

These results build on previous studies providing evidence in favor of ΔR for integrating longitudinal variations of blood biomarkers in NSCLC patients. Khorrami et al^16^ described a high delta radiomic score resulting from the linear combination of selected features to be associated with an increased mortality risk in 139 NSCLC patients treated with ICIs, but no systemic indices were included in the models. In a recent study by Cousin et al,^18^ a Cox-regression delta radiomic model showed a better performance than clinical information for OS prediction in advanced or recurrent NSCLC. In that cohort, however, clinical predictors were retrieved from a single time point evaluation and did not enrich radiomics models.^18^ Gong et al^20^ demonstrated a role for ΔR in predicting OS and progression-free survival in 224 retrospectively collected NLSC cases, yet they did not explore possible blood biomarkers. Our study uncovered the combined longitudinal variations of imaging with SI indices as a promising tool to predict survival in NSCLC. Moreover, in keeping with previous data, the current evidence points attention toward a potential ΔR superiority compared with single-time-point radiomic models for risk stratification purposes.^16,18,20^

Our ΔR signature included lesion shape and gray intensity descriptors, potentially enabling a comprehensive assessment of lesion behavior beyond tumor diameters. An in-depth comparison with previous ΔR signatures is inherently limited by methodological differences, such as study population selection, model building, and objectives. The disease progression, which has been explored in most ΔR studies, was not evaluated to avoid incorporation biases, as it includes information from the analyzed images by definition.^29^ Furthermore, we extracted the CT features only from the primary lesion, which was shown to be more predictive than multiple lesions-derived signatures, saving time from segmentation processing.^18,19^ It is worth emphasizing that our approach is intended to be preliminary and needs to be interpreted cautiously. Further evidence is warranted to optimize ΔR workflow and improve the explainability of its predictive role in NSCLC patients treated with ICIs.

Systemic inflammatory indices were confirmed as clinically valuable parameters in ICI-treated NSCLC, in agreement with the notion that cancer-associated inflammatory state conditions poor survival.^30^ Interestingly, in the longitudinal model, LDH but not dNLR was retained for predicting the risk of death. It is well-known that LDH catalyzes pyruvate to produce lactic acid, while dNLR considers variations in white blood cells and neutrophil count.^31^ The different yet potentially complementary role of these indices in the survival stratification of NSCLC patients is likely to be driven by distinct intrinsic trajectories. Although our analyses enable speculating about LDH reflecting the dynamic of tumor-host interaction better than dLNR, the study was not designed to explore causative links and needs further in-depth investigations, ideally taking into account the potential confounding role of concomitant drugs (eg, steroids) that may affect dNLR discriminative ability.^32^

Host-related factors are recognized to impact treatment response in the ICI-driven scenario.^33,34^ The number and sites of metastatic lesions have been reported as a determinant of impaired ICIs activity in advanced NSCLC.^33,35^ Among the variables selected by regression analyses, bone metastases at baseline CT were significantly associated with an increased risk of death. This result is in keeping with prior studies that ascribed the negative prognostic role of bone involvement to a putative immunosuppressive role exerted by the marrow compartment and its immunoregulatory function.^36,37^ Nevertheless, it remains difficult to fully determine the reason underlying the superiority of bone metastasis over other clinical and laboratory variables in the present survival analysis. Irrespective of the magnitude of the increased mortality risk carried out by osseous involvement in our cohort, the current findings highlight the relevance of an approach where longitudinal changes in imaging and clinical-laboratory parameters are likely to integrate rather than overcome baseline information, which remains essential for elaborating prognostic strategies in NSCLC patients.

Some limitations of the present study should be acknowledged. First, the sample size was relatively small, and we did not test our signatures in an independent population, which calls for caution about the generalizability of the results. This may be particularly relevant as the included CT images were obtained from different scanners and uncontrolled acquisition and reconstruction parameters, possibly affecting the models’ performances in external cohorts; however, to date, there are no established prevention methods for addressing this possible source of bias in retrospective radiomic studies, and the optimum way to mitigate the effects of image acquisition and processing variability on radiomic models pend future research.^29^ Second, we did not consider PD-L1 expression in the model building due to the limited data availability. Third, the patient population heterogeneity hampered comprehensive stratification analyses to control for all possible confounding. Last, prospective RECIST 1.1 rather than iRECIST criteria were considered for our analyses. However, pseudoprogression is an infrequent occurrence, which may mitigate eventual impacts on survival prognostication in our study.^38,39^

In conclusion, ΔR has shown the potential to improve OS prediction compared with a single-point-radiomic approach in NSCLC patients treated with ICIs. The integration of ΔR with a longitudinal assessment of clinical and laboratory data, notably systemic inflammatory indices, further improves NSCLC survival stratification, thus contributing to NSCLC patient risk individualization.

## Supplementary Material

SUPPLEMENTARY MATERIAL
