# Supplementary figures and images for: Longitudinal Changes of CT-radiomic and Systemic Inflammatory Features Predict Survival in Advanced Non–Small Cell Lung Cancer Patients Treated With Immune Checkpoint Inhibitors
Source: J Thorac Imaging. 2024 Nov 25;40(1):e0801. doi: 10.1097/RTI.0000000000000801 (PMC11654449; doi:10.1097/RTI.0000000000000801)

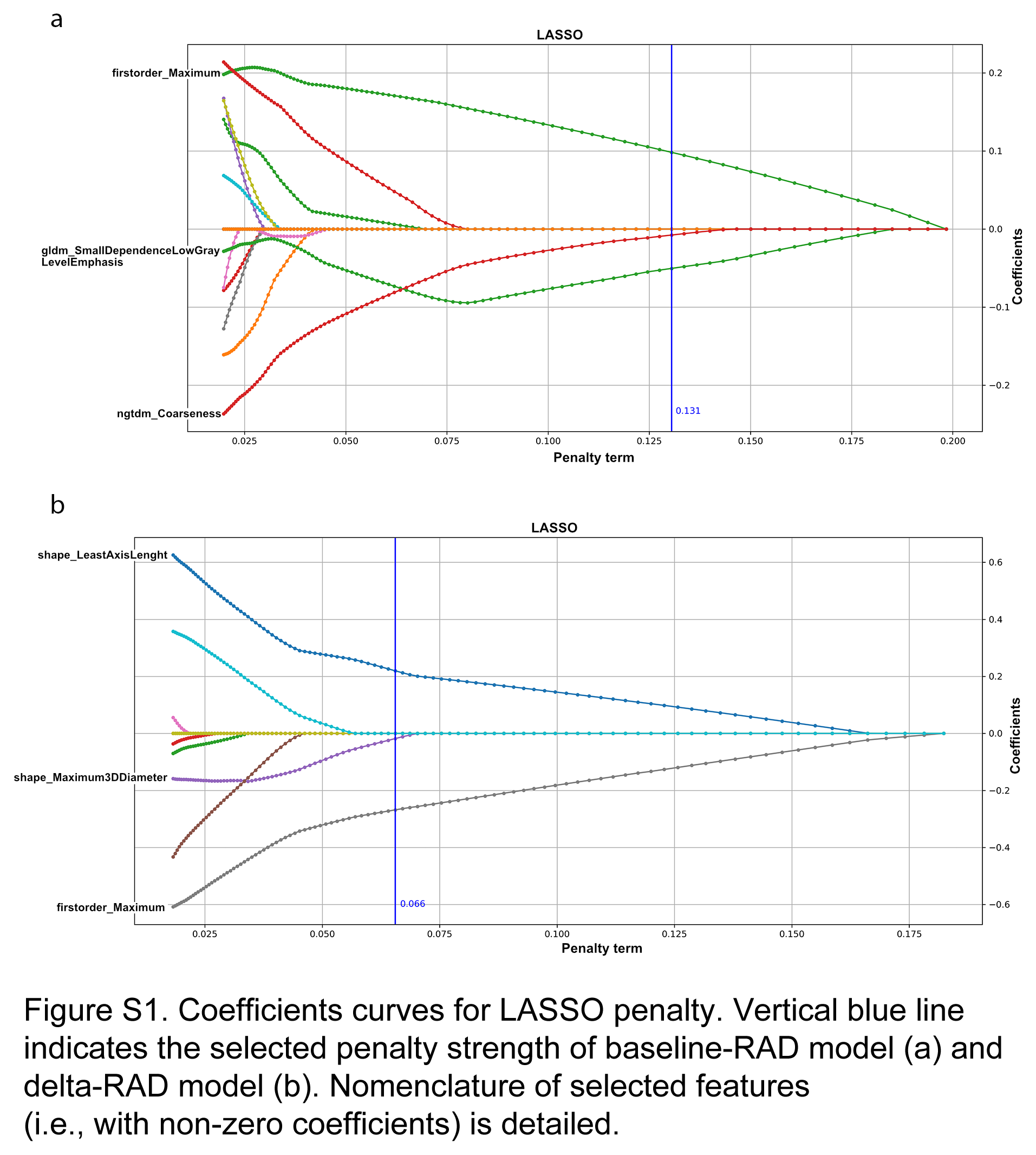

Supplement: SUPPLEMENTARY MATERIAL [file rti-40-e0801-s001.tif]

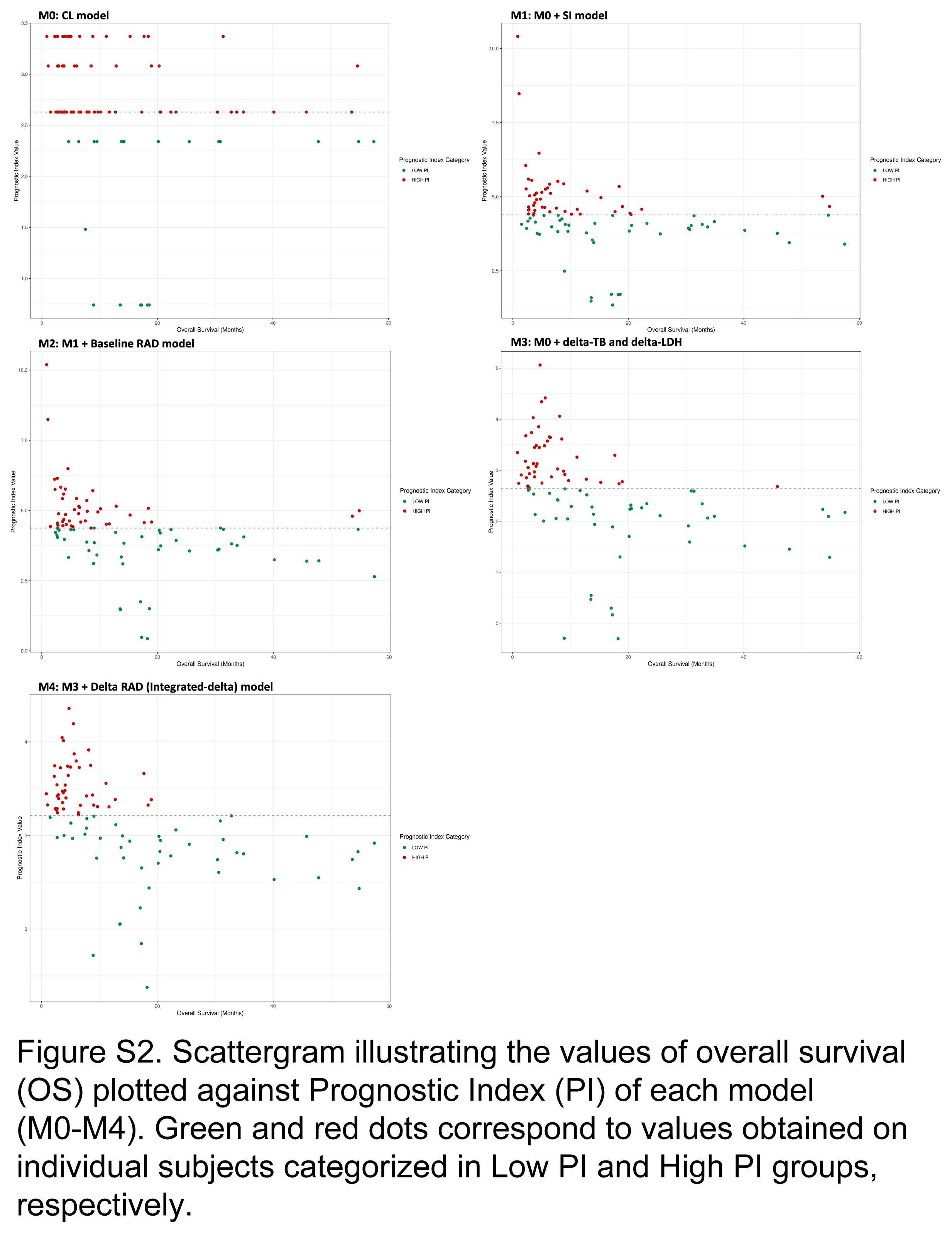

Supplement: SUPPLEMENTARY MATERIAL [file rti-40-e0801-s002.tif]
